# The suicide assessment scale: Psychometric properties of a Norwegian language version

**DOI:** 10.1186/1756-0500-5-417

**Published:** 2012-08-07

**Authors:** Bjørn Odd Koldsland, Lars Mehlum, Liv Solrunn Mellesdal, Fredrik A Walby, Lien M Diep

**Affiliations:** 1 Vestre Viken Health Trust, Ringerike Hospital psychiatric out-patient clinic, Oslo, Norway; 2 National Centre for Suicide Research and Prevention, Institute of Clinical Medicine, University of Oslo, Oslo, Norway; 3 Psychiatric Division, Haukeland University Hospital, Bergen, Norway; 4 Department of Psychiatry, Diakonhjemmet Hospital, Oslo, Norway; 5 Oslo University Hospital, Oslo, Norway

## Abstract

**Background:**

Rating scales are valuable tools in suicide research and can also be useful supplements to the clinical interview in suicide risk assessments. This study describes the psychometric properties of a Norwegian language version of the Suicide Assessment Scale Self-report version (SUAS-S).

**Methods:**

Participants were fifty-two patients (mean age = 39.3 years, SD = 10.7) with major depression (53.8%), bipolar disorder (25.0%) and/or a personality disorder (63.5%) referred to a psychiatric outpatient clinic. The SUAS-S, the screening section of the Beck Scale for Suicidal Ideation (BSS-5), the Beck Depression Inventory (BDI), Beck’s Hopelessness Scale (BHS), the Symptom Check-List-90 R (SCL-90R) and the Clinical Global Impression for Severity of Suicidality (CGI-SS) were administered. One week later, the patients completed the SUAS-S a second time.

**Results:**

Cronbach’s alpha for SUAS-S was 0.88 and the test–retest reliability was 0.95 (95% CI: 0.93– 0.97). SUAS-S was positively correlated with the BSS-5 (r = 0.66; 95% CI: 0.47–0.85) for the study sample as a whole and for the suicidal (r = 0.52) and non-suicidal groups (r = 0.50) respectively. There was no difference between the SUAS-S and the BSS-5 in the ability to identify suicidality. This ability was more pronounced when the suicide risk was high. There was a substantial intercorrelation between the score on the SUAS-S and the BDI (0.81) and the BHS (0.76). The sensitivity and specificity of the SUAS-S was explored and an appropriate clinical cut-off value was assessed.

**Conclusions:**

The study revealed good internal consistency, test–retest reliability and concurrent validity for the Suicide Assessment Scale Self-report version. The discriminatory ability for suicidality was comparable to that of the BSS-5.

## Background

Assessment of suicide risk is one of the most challenging tasks that clinicians working in mental health care face. The difficulty of the task derives from problems with the definition of suicidality [1], the fact that suicide risk may vary considerably with time and across contexts, and, above all, the low incidence of completed suicide [2]. To date, the clinical interview has been regarded as the gold standard for suicide risk assessment [3], but even though it is very resource demanding it has relatively low sensitivity and specificity. In a clinical context adding a self-report questionnaire could provide information that would otherwise remain undetected or undisclosed during the clinical interview [4]. Furthermore, self-report instruments could be a useful supplementary low-cost method to secure repeated measurements of suicide risk in contexts where this is regarded important. Several suicide risk assessment questionnaires have been developed for research and clinical purposes [5]. These instruments are highly heterogeneous. The Suicide Assessment Scale (SUAS) was developed by Stanley and co-workers [6] as a clinician-administered rating scale, and was further developed by Nimeus et al. [7,8] as a self-report instrument (SUAS-S) [7,9]. The SUAS scale was designed to tap both explicit suicide risk factors and information on indirect dimensions relevant to suicidality, such as affective instability and poor impulse control. The aim of this study was to investigate the psychometric properties of a Norwegian language version of the SUAS-S (Additional file 1).

## Methods

### The scale

The SUAS-S is a 20-item self-report rating scale measuring the patient’s attitude towards suicide, suicide-related behaviour and suicidal ideation on the day of reporting and during the previous seven days. Each item is scored in the range of 0–4 on a Likert-type scale and resulting in a scale sum score with a range of 0–80. The scale is designed to measure levels of suicidality and to be sensitive to temporal changes in suicide-related symptoms [6]. The SUAS-S items cover five thematic areas [6,8]: “affect” (items 1, 2, 9, 12 and 13), “bodily states” (items 3, 8 and 10), “control and coping” (items 6, 7, 11 and 15), “emotional reactivity” (items 4, 5 and 14) and “suicidal thoughts and behaviour” (items 16–20). The original SUAS-S scale has been found to possess good concurrent validity compared with the interview version [9].

### Sample

Fifty-five patients consecutively referred to a psychiatric outpatient clinic from January 2008 until January 2009, were asked to participate in the project. Fifty-three (96%) of the patients provided informed consent and were included in the study. Of these, one patient dropped out before all scales were completed and was not included in the further analysis. Patients with an on-going substance abuse and/or psychotic disorder were not eligible to participate in the study. Patients were recruited from a non-emergency setting.

### Ratings

Deliberate self-harm (DSH) was defined as “an act with non-fatal outcome in which an individual deliberately initiated behaviour with the intention to cause self-harm, for example, self-cutting or jumping from a height, by ingestion of an illicit drug, a non-ingestible substance or an excess of a prescribed substance”[10,11]. Suicide attempt (SA) was defined as “a potentially self-injurious behaviour with a nonfatal outcome, for which there is evidence that the person intended, at some level, to kill himself/herself”[11]. The Clinical Global Impression of Severity of Suicidality Scale (CGI-SS) [12] was used in the baseline assessment to provide an overall clinician rated measure of the clinical risk of suicidality in each participant. The CGI-SS has five levels of severity of suicidality; 1 = not at all suicidal, 2 = mildly suicidal, 3 = moderately suicidal, 4 = severely suicidal, and 5 = attempted suicide. Interviews yielding these scores were made independent of and blind to the participants' SUAS-S scores. Study subjects were categorically classified as “non-suicidal” if they received scores 1 or “suicidal” if they were scored 2–5 on the CGI-SS. The baseline assessment also included measuring depressive symptoms with the Beck Depression Inventory (BDI) [13], feelings of hopelessness with Beck’s Hopelessness Scale (BHS) [14], general psychiatric symptoms with the Symptom Check List-90 R (SCL-90 R) [15], suicidal ideation with the five items screening section of Beck’s Scale for Suicidal Ideation (BSS-5) [16] and general functioning level with the Global Assessment of Functioning (GAF) [17]. The SUAS-S was administered a second time one week after the first administration. Psychiatric diagnoses were made at baseline using the Mini International Neuropsychiatric Interview (M.I.N.I.) [18] for DSM-IV axis I disorders and the Structured Clinical Interview for DSM-IV Personality Disorders (SCID-II) [19] for axis II disorders. Interviews and diagnoses were all made by the first author, an experienced psychiatrist. In cases where it was unclear whether criteria for a specific diagnosis were met, the case was discussed with the second author (a professor of psychiatry) and settled by consensus. In cases where the patients had more than one DSM IV axis I or axis II diagnosis, only the main diagnosis on each axis was considered in the analyses. Diagnostic data were collected to provide a good description of this clinical sample. The study was, however, not designed to study the SUAS-S properties in diagnostic subgroups.

### Statistical analysis

Means and standard deviations, or medians and quartiles, were given for sum scores. The chi-squared test was used to assess differences in distribution between the groups for categorical variables. Differences in sum scores of two independent groups were tested by two-sample *t*-test or Mann–Whitney *U* test. Internal consistency was computed as the Cronbach’s alpha coefficient, and test–retest reliability was measured with Intra Class Correlation (ICC) with 95% Confidence Intervals (CIs). Associations between the sum scores on the SUAS-S and BSS-5, and their precision, were estimated by Pearson’s or Spearman’s correlation coefficient and bootstrapped 95% CI with 10,000 replications. Discriminatory ability of suicidality for the SUAS-S and BSS-5 sum scores was examined and compared through the use of nonparametric receiver operating characteristic (ROC) curve analyses. The tests were two-sided, and the significance level was set at 0.05. The analyses were performed with STATA 11 [20] and the graphs were made in R 2.11.1 [21].

### Ethics

The study was approved by the Regional Committee for Medical Research Ethics, South-East Norway. All patients gave written informed consent.

## Results

### Sample characteristics

The sample consisted of 33 (63.5%) females and 19 males (36.5%) with a mean age of 39.3 years (SD = 10.7, range = 21–62). Thirty-four patients (65.4%) had been in psychiatric treatment in total for more than five years, and 11 (21.2%) for less than a year. The mean GAF score was 48.6 (SD = 4.6); there were no gender differences. The mean SCL-90-R score was 1.5 (SD = 0.7), with no significant gender differences. Twenty-two (42.3%) of the patients, 16 females and six males, reported a history of DSH. In 15 (68.2%) of these cases, the criteria for a SA were met. Five patients reported having had a DSH episode during the last six months. The median number of DSH episodes per patient was one, with a range of 0–30.

All but one patient fulfilled the criteria for at least one axis I disorder. Major Depressive Disorder (MDD) was diagnosed in 28 patients (53.8%), Bipolar Disorder type II in 13 cases (25.0%) and Dysthymia in 4 cases (7.5%). These diagnostic groups were merged into one group, “Affective Disorders” (AD) (n = 45). The residual group (n = 7) was labelled “Other Axis I Disorders”. Thirty-three patients (63.5%) had an axis II diagnosis, of whom five (9.6%) had a Cluster A disorder, 11 (21.2%) a Cluster B disorder, 15 (28.8%) a Cluster C disorder, and two patients had Personality Disorder Not Otherwise Specified (PF-NOS).

### SUAS-S scores

The mean SUAS-S score was 31.0 (SD = 9.3) in females and 28.1 (SD = 12.7) in males. No significant correlation was found between age and the SUAS-S score. Patients with AD had a significantly higher median SUAS-S score (median = 31.0, quartiles = 24.5–39.0) than patients with other axis I disorders (median = 19, Q1-Q3 quartiles = 15–31), (p = 0.018).

### Psychometric properties of the SUAS-S

Internal consistency, as measured by Cronbach’s alpha coefficient, was found to be 0.88 (95% CI: 0.82–0.92). The test–retest reliability expressed by the ICC was 0.95 (95% CI: 0.93–0.97). Concurrent validity was examined by pairwise correlations with 95% CIs between SUAS-S, BSS-5, BDI and BHS-scores for all patients, as shown in Table 1.

**Table 1:** Intercorrelations with 95% CIs between scores on the SUAS-S, BSS-5, BDI and BHS

	**SUAS-S**	
	** Overall (n = 52)**	**P**^**a**^
BSS-5	0.66 (0.47–0.85)	< 0.001
BDI	0.81 (0.70–0.92)	< 0.001
BHS	0.76 (0.61–0.92)	< 0.001

^a^P-values of pairwise correlations in the overall.

**Figure 1:**
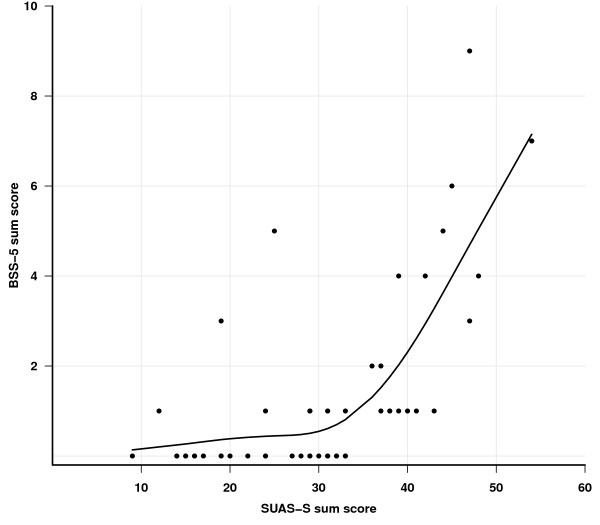
Co-variation between BSS-5 and SUAS-S sum scores shown as a solid line (all patients included).

SUAS-S was generally strongly correlated with these measures, but somewhat weaker with BSS-5. The non-linear co-variation between the BSS-5 and SUAS-S scores is more clearly shown in Figure 1. The association implies a tendency for scores on the BSS-5 to increase only after the SUAS-S scores have exceeded 30. The mean and median SUAS-S and BSS-5 scores as well as the BDI and BHS scores were significantly higher in patients classified according to the interview based CGI-SS-score as “suicidal” (n = 25) than in patients classified as “non-suicidal” (n = 27) (Table 2).

**Table 2:** Test score means with standard deviations and medians with quartiles and effect size in suicidal and non-suicidal patients

	**Suicidal (n = 25)**	**Non-suicidal (n = 27)**	**Effect size**	**P**
	**Mean ± SD**	**Mean ± SD**		
SUAS-S	33.7 ± 9.7	25.6 ± 9.3	0.85	0.003^a^
BSS-5*	1.0 (1.0–4.0)	0.0 (0.0–0.3)	1.07**	< 0.001^b^
BDI	23.0 ± 11.0	14.2 ± 8.8	0.89	0.003^a^
BHS*	11.0 (6.0–16.0)	3.0 (2.8–9.0)	0.92**	0.005^b^

*Median (Q1-Q3), **Assuming normally distributed data.

^a^Two independent samples *t*-test, ^b^Mann–Whitney *U* test.

The sensitivity and specificity of the SUAS-S was explored as tabulated in Table 3.

**Table 3:** Sensitivity, specificity and predictive probability of SUAS-S and BSS-5 cut-off points for detecting suicidality

	**Cut-off**	**Sens**^**1**^**(%)**	**Spec**^**2**^**(%)**	**PPV**^**3**^**(%)**	**NPV**^**4**^**(%)**
SUAS-S	≥ 28	80	52	61	74
	≥ 29	80	56	63	75
	≥ 30	68	63	63	68
	≥ 31	68	67	65	69
BSS-5	≥ 0	100	0	77	81
	≥ 1	80	78	77	40
	≥ 2	36	93	82	61
	≥ 3	32	96	78	52

^1^Sensitivity, ^2^Specificity, ^3^Positive predictive probability, ^4^Negative predictive probability.

A cut-off value of 29 on the SUAS-S scale yielded a sensitivity of 80% and specificity of 56%. The negative and positive predictive probabilities of this chosen cut-off point were 75% and 63%, respectively.

### Identification of suicidality, depression and hopelessness

Descriptive and association analyses of the five areas of the SUAS-S scale, with a categorical classification as suicidal on the CGI-SS scale as the dependent variable, showed that the sum scores of the area “suicidal thoughts and behaviour” (SUAS-S items 16–20) was strongly associated with this variable (p < 0.001). The other three areas, “bodily states”, “control and coping” and “emotional reactivity” were not significantly associated with this dependent variable. The results of analyses are shown in Table 4.

**Table 4:** Mean scores with standard deviations, medians with quartiles and effect size for the five SUAS-S thematic areas

	**Suicidal (n = 25)**	**Non-suicidal (n = 27)**	**Effect size**	**P**
	**Mean ± SD**	**Mean ± SD**		
Affect	10.2 ± 2.6	8.7 ± 2.4	0.60	0.030^a^
Bodily States	5.0 ± 2.0	4.4 ± 2.1	0.29	0.364^a^
CaC	6.1 ± 2.0	5.2 ± 2.1	0.44	0.106^a^
ER	5.0 ± 2.3	4.6 ± 2.3	0.17	0.526^a^
STaB*	8.0 (5.0 - 11.0)	1.0 (0.0 - 4.0)	1.15**	< 0.001^b^

CaC: Control and coping, ER: Emotional reactivity, STaB: suicidal thoughts and behaviour.

^a^Two independent sample *t*-test, ^b^Mann-Whitney *U* test.

*Median (Q1-Q3), **Assuming normally distributed data.

Pairwise associations between the BSS-5, depression, hopelessness and the sum scores of the five areas are shown in Table 5.

**Table 5:** Intercorrelations with 95% CIs between the five areas of SUAS-S, BSS-5, BDI and BHS sum scores

	**BSS-5**	**BDI**	**BHS**
Affect	0.52 (0.27-0.73)	0.75 (0.61-0.84)	0.76 (0.60-0.87)
Bodily States	0.22 (-0.07-0.49)	0.50 (0.27-0.69)	0.29 (0.00-0.54)
CaC	0.40 (0.11-0.64)	0.57 (0.32-0.74)	0.55 (0.31-0.73)
ER	0.36 (0.09-0.60)	0.65 (0.43-0.81)	0.60 (0.37-0.76)
StaB	0.81 (0.69-0.89)	0.68 (0.50-0.80)	0.67 (0.46-0.81)

CaC: Control and coping, ER: Emotional reactivity, STaB: Suicidal thoughts and behaviour.

Correlation analyses showed that the SUAS-S areas of “affect” and “suicidal thoughts and behaviour” and “emotional reactivity” were strongly and significantly associated with depression and hopelessness (r ≥ 0.6, p < 0.001). The “bodily states” area was significantly correlated with depression (expressed by the BDI sum score) but not with hopelessness (expressed by the BHS sum score). The association was strongest between the sum scores of the area “suicidal thoughts and behaviour” and BSS-5 (r ≥ 0.8, p < 0.001). The results of correlation analyses are shown in Table 5.

**Figure 2:**
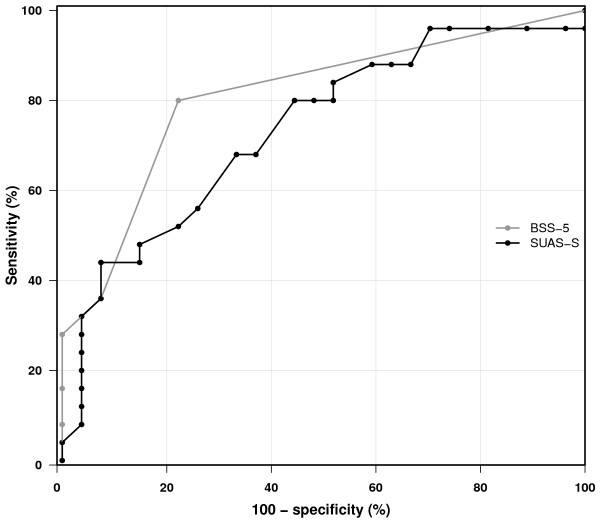
Receiver operating characteristic curves for SUAS-S with BSS-5 to diagnose suicidality.

Figure 2 shows receiver operating characteristic (ROC) curves of the sensitivity (true positive rate) versus 1–specificity (the true negative rate) of the test. The discriminatory performance of the test is summarized by the area under the ROC curve. The larger the area under the ROC curve, the better the discrimination. For a given cut-off point, the closer both sensitivity and specificity (i.e., 1–specificity, close to zero) are to 1, the better the discriminatory performance. As shown in the figure, no significant psychometric differences were found in the ROC analyses for the SUAS-S 0.74 (95% CI: 0.60–0.87) or BSS-5 0.81 (95% CI: 0.70–0.92), (p = 0.238).

## Discussion

The psychometric properties of the Norwegian language version of the SUAS-S in a non-emergency psychiatric outpatient setting were found to be good, with internal consistency and test–retest reliability properties comparable to the original version. Likewise, the construct validity was found to be favourable. Of the five areas in the SUAS-S, the “suicidal thoughts and behaviour” subscale was significantly and strongly associated with the clinician’s global assessment of suicidality. The findings of a strong correlation between the SUAS-S and BSS-5 (0.66), and no significant difference between the ROCs for these two scales (Figure 2), suggests that the SUAS-S is able to identify suicidality equally well as the BSS-5. By indicating a cut-off-value of 29, a sensitivity of 80% and a specificity of 56% were attained (Table 3). In the context of clinical suicide assessment, these specificity and sensitivity levels may be regarded as adequate. That the SUAS-S scores were highly correlated with the BDI-scores in the overall sample and the subgroups, indicate that SUAS-S may have the benefit of tapping depression in addition to suicidality. It is important to note that whereas the BSS-5 is a short screening instrument, the SUAS-S taps several additional clinically important dimensions relevant to assessment and management of suicidal patients in a clinical context. The SUAS-S seems also, as evident from Figure 1, to be sensitive to lower symptom levels which may well be of value when monitoring the clinical course with repeated measurements to evaluate treatment response and detect signs of relapse.

### Limitations

The limited number of patients excluded the possibility to study the SUAS-S properties in more narrow diagnostic subgroups. The selection criteria applied in this study with exclusion of on-going substance abuse and psychotic disorders limits the external validity of findings to non-psychotic and non-abusing patients in non-emergency psychiatric evaluation settings.

## Conclusions

The Norwegian language version of the SUAS-S was found to possess psychometric properties equal to the original version and seems to be a valid and reliable instrument in the assessment of suicide risk and depression among patients in the mental health care system. The SUAS-S also has the advantage of open access with no copyright costs.

## Competing interests

All authors declare that they have no conflicts of interest.

## Authors’ contributions

BOK and LM designed the study. BOK collected the data and LMD performed the statistical analyses and contributed to the writing of the manuscript. BOK, LM, LSM and FAW wrote the manuscript and all authors approved the final version.

## Authors’ information

B.O.Koldsland MD is a senior psychiatrist at Vestre Viken Health Trust, Ringerike Hospital psychiatric outpatient clinic, and affiliated with the National Centre for Suicide Research and Prevention, University of Oslo. L. Mehlum MD PhD is a professor and head of the National Centre for Suicide Research and Prevention, Institute of Clinical Medicine, University of Oslo. L.S. Mellesdal RN MSc is a research fellow in the Psychiatric Division of Haukeland University Hospital, Bergen*.* F. A. Walby MA is a researcher at the National Centre for Suicide Research and Prevention, Institute of Clinical Medicine, University of Oslo and chief psychologist in the Department of Psychiatry, Diakonhjemmet Hospital, Oslo. L.M. Diep MSc is a statistician at Oslo University Hospital, the National Centre for Suicidal Research and Prevention at University of Oslo, and the Institute for Community Medicine at University of Oslo, Norway.

## Supplementary Material

Additional file 1 **Appendix.** SUAS. Click here for file
