# Supplementary material for: The suicide assessment scale: Psychometric properties of a Norwegian language version
Source: BMC Res Notes. 2012 Aug 7;5:417. doi: 10.1186/1756-0500-5-417 (PMC3504573; doi:10.1186/1756-0500-5-417)
Supplement: Additional file 1 — Appendix. SUAS. [file 1756-0500-5-417-S1.docx]

| **SUAS**  **Dato for utfylling … / … - ….** | |
| --- | --- |
| **Selvutfylling av pasient** | |
| **Instruksjon** | |
| Nå kommer noen spørsmål om hvordan du har det med deg selv og dine omgivelser. Vær snill å lese nedenforstående grupper av påstander nøye. Sett ring rundt den påstanden i hver gruppe som best beskriver hvordan du har følt deg den siste uken, inkludert i dag. Vær nøye med å lese alle påstander i hver gruppe før du bestemmer deg for hva du skal sette ring rundt. Du kan eventuelt oppleve at noen grupper av påstander ikke gjelder deg. Da svarer du ”0” Noen av spørsmålene ligner mye på spørsmål du allerede har svart på, men det er fint om du likevel vil forsøke å svare på dem. | |

| **1.** |
| --- |

| Jeg kan være både glad og trist, alt etter omstendighetene. | **0** |
| --- | --- |
| Jeg er for det meste positiv og glad, men kan ha perioder der jeg føler meg motløs. | **1** |
| Jeg er ofte nedstemt, selv om lysere stunder forekommer. | **2** |
| Jeg er nesten alltid nedstemt og fortvilet, bedre stunder er sjeldne. | **3** |
| Livet mitt er totalt ødelagt av den dypeste fortvilelse og lidelse. | **4** |

| **2.** | | |
| --- | --- | --- |
| Jeg blir sjelden provosert. | **0** |  |
| Jeg har lettere for å bli provosert eller sint enn det som er vanlig for meg. | **1** |  |
| Jeg kjenner meg ofte irritert eller provosert uten noen direkte årsak. | **2** |  |
| Jeg kjenner meg nesten alltid irritert og iblant skikkelig sint uten noen direkte årsak. | **3** |  |
| Jeg er alltid veldig irritert og sint, uten at det egentlig er noen direkte årsak. Jeg har store problemer med å beherske meg. | **4** |  |

| **3.** | |
| --- | --- |
| Jeg har ingen problemer med å greie mine daglige aktiviteter og blir sjelden trøtt. | **0** |
| Jeg ivaretar mitt daglige arbeid, men blir ofte trøtt. | **1** |
| Jeg har iblant problemer med å ivareta mine daglige aktiviteter og må ofte ta pauser for å hvile. | **2** |
| Jeg er nesten alltid svært trøtt og sliten. Må ofte avbryte det jeg holder på med. | **3** |
| På grunn av en overveldende trøtthet er jeg helt ute av stand til å gjøre noe som helst. | **4** |

| **4.** | |  |
| --- | --- | --- |
| Jeg er ikke spesielt vár for å bli irettesatt eller for kritikk. | **0** | |
| En sjelden gang kan jeg føle meg avvist eller personlig forulempet hvis noen irettesetter eller kritiserer meg. | **1** | |
| Jeg har lettere enn vanlig for å føle meg avvist eller personlig forulempet hvis jeg blir irettesatt eller kritisert. | **2** | |
| Jeg føler meg svært ofte dypt såret og personlig forulempet over irettesettelse eller kritikk. | **3** | |
| Mine omgivelser forsøker bevisst å skade meg ved irettesettelse og kritikk. | **4** | |

| **5.** | |  |
| --- | --- | --- |
| Jeg har bra og regelmessig kontakt med mine venner og slektninger. | **0** | |
| Jeg har bra kontakt med mine venner og slektninger, men sjeldnere enn før. | **1** | |
| Jeg har for tiden bare kontakt med noen få av mine nærmeste venner og slektninger. | **2** | |
| Det er bare en og annen venn jeg snakker med en gang iblant. | **3** | |
| Jeg orker ikke å ha med andre mennesker å gjøre og lever helt isolert. | **4** | |

| **6.** | |  |
| --- | --- | --- |
| Jeg håndterer følelsesmessige problemer på en tilfredsstillende måte. | **0** | |
| Jeg kan en sjelden gang ha vansker med å hanskes med følelsesmessige problemer. | **1** | |
| Jeg har begrenset evne til å se alternative løsninger på mine følelsesmessige problemer. | **2** | |
| Jeg ser sjelden eller aldri løsninger på mine følelsesmessige problemer. | **3** | |
| Jeg føler meg helt ute av stand til bare å tenke på det minste følelsesmessige problem. | **4** | |

| **7.** | |  |
| --- | --- | --- |
| Jeg stoler på meg selv og mine beslutninger. | **0** | |
| En sjelden gang kan jeg bli usikker på min evne til å håndtere min egen situasjon. | **1** | |
| Jeg er oftere overlatt til skjebnen eller omgivelsene, enn til min egen mestring. | **2** | |
| Jeg tar svært sjelden egne beslutninger. Derimot er jeg overlatt til omgivelsene eller skjebnen. | **3** | |
| Jeg er helt kontrollert av skjebnen eller omgivelsene, uten innflytelse på min egen tilværelse. | **4** | |

| **8.** | |  |
| --- | --- | --- |
| Jeg føler meg avslappet. | **0** | |
| Jeg har større problemer enn vanlig med å slappe av. | **1** | |
| Jeg er ofte ubehagelig anspent i hele kroppen. | **2** | |
| Jeg er nesten aldri avslappet. Muskelspenning og andre fysiske ubehag dominerer. | **3** | |
| Jeg er aldri avslappet, derimot lider jeg konstant av besværlig og plagsom muskelspenning. | **4** | |

| **9.** | |
| --- | --- |
| Jeg føler meg rolig og lite engstelig. | **0** |
| Jeg har lettere enn vanlig for å bli urolig. | **1** |
| Jeg blir lett engstelig og urolig og har lett for å overdrive mine bekymringer. Likevel dominerer de rolige stundene. | **2** |
| Jeg føler meg sjelden eller aldri rolig. Bekymring og frykt for både nuet og min fremtid gjør meg urolig. | **3** |
| Jeg har stadig sterk angst og følelse av ulyst. Plages av bekymring og frykt. | **4** |

| **10.** | |  |
| --- | --- | --- |
| Jeg føler meg kroppslig frisk. | **0** | |
| Jeg er iblant urolig over min kroppslige helse, men kan likevel lett slå tankene fra meg. | **1** | |
| Jeg grubler og uroer meg ofte over min kroppslige helse. Jeg må iblant be mine mine omgivelser om hjelp for å bli kvitt tankene. | **2** | |
| Jeg har ganske sikkert en fysisk sykdom. På tross av at jeg mange ganger har nevnt det for mine omgivelser, er det ingen som vil høre på meg. | **3** | |
| Jeg har en alvorlig fysisk sykdom, men mine omgivelser vil ikke tro på meg. | **4** | |

| **11.** | |  |
| --- | --- | --- |
| Jeg tenker meg om før jeg tilfredsstiller mine ønsker og behov. | **0** | |
| En sjelden gang hender det at jeg handler uten å tenke på konsekvensene. | **1** | |
| Jeg har ofte problemer med å holde igjen mine impulsive ønsker og behov, men tanken på eventuelle konsekvenser kan hindre meg. | **2** | |
| Jeg handler etter ønsker og behov, oftest helt uten tanke på eventuelle konsekvenser. | **3** | |
| Alt jeg gjør skjer helt impulsivt og jeg bryr meg ikke om eventuelle konsekvenser. | **4** | |
|  |  | |
| **12.** | |  |
| Jeg har god selvtillit. | **0** | |
| En sjelden gang kan jeg miste troen på mine egne evner. Jeg kan likevel korrigere meg selv. | **1** | |
| På tross av at jeg vanligvis stoler på meg selv, så har jeg tilbakevendende opplevelse av mislykkethet og jeg føler meg stadig usikker på mine evner. | **2** | |
| Jeg føler meg nesten alltid verdiløs og mislykket, og jeg tviler på at den følelsen kan endres til det bedre. | **3** | |
| Jeg er helt mislykket og verdiløs og det finnes ikke håp om bedring. | **4** | |

| **13.** | |  |
| --- | --- | --- |
| Jeg ser lyst på fremtiden. | **0** | |
| En sjelden gang kan jeg se mørkt og pessimistisk på min fremtid. | **1** | |
| Jeg ser ofte mørkt og pessimistisk på min fremtid. Håpefulle tanker forekommer sjelden. | **2** | |
| Jeg har bare mørke og pessimistiske forventninger om min fremtid. Positive tanker forkommer aldri. | **3** | |
| Jeg føler meg helt håpløs og fortvilet. Hva som helst kan hende meg. | **4** | |

| **14.** | |  |
| --- | --- | --- |
| Jeg er interessert og engasjert i mine omgivelser. | **0** | |
| Jeg synes iblant at det er vanskelig å engasjere meg i mine omgivelser. | **1** | |
| Jeg synes ofte at det er svært vanskelig å interessere meg for og engasjere meg i mine omgivelser. | **2** | |
| Jeg føler meg oftest helt likegyldig og uinteressert, selv overfor mine nære venner og slektninger. | **3** | |
| Jeg plages av min totale mangel på interesse, til og med overfor mine aller nærmeste. | **4** | |

| **15.** | |  |
| --- | --- | --- |
| Jeg blir bare irritert eller frustrert når det finnes grunn til det. | **0** | |
| Jeg har en sjelden gang blitt irritert eller frustrert over bagateller. | **1** | |
| Jeg har flere ganger blitt irritert eller frustrert over små bagateller. | **2** | |
| Jeg er stadig irritert eller frustrert uten noen grunn. | **3** | |
| Jeg er alltid irritert eller frustrert uten noen som helst grunn. | **4** | |
|  |  | |
| **16.** | |  |
| Jeg har mange gode grunner til å leve. | **0** | |
| Jeg har iblant negative tanker om meningen med livet, men jeg er overbevist om å fortsette å leve. | **1** | |
| Jeg er til stadighet usikker på om jeg ønsker å leve, men de positive sidene ved livet dominerer. | **2** | |
| Mine grunner til fortsatt å leve er få og tvilsomme og jeg føler meg usikker på om jeg ønsker å leve. | **3** | |
| Jeg ser ingen grunn til fortsatt å leve. | **4** | |

| **17.** | |  |
| --- | --- | --- |
| Jeg har ingen ønsker om å dø. | **0** | |
| Jeg kan en sjelden gang tenke på min egen død, men viljen til å leve er stor. | **1** | |
| Tanker om min egen død forekommer og kan iblant kjennes befriende. | **2** | |
| Døden innebærer for meg noe positivt og mitt ønske om å leve er svakt. | **3** | |
| Jeg lengter etter og ønsker at jeg var død. | **4** | |

| **18.** | |  |
| --- | --- | --- |
| Jeg har ingen selvmordstanker. | **0** | |
| Jeg har en sjelden gang hatt selvmordstanker. | **1** | |
| Jeg har ved flere anledninger hatt selvmordstanker. | **2** | |
| Jeg har svært ofte selvmordstanker. | **3** | |
| Jeg har selvmordstanker hele tiden. | **4** | |

| **19.** | |  |
| --- | --- | --- |
| Jeg har ingen selvmordstanker. | **0** | |
| Hvis jeg gjorde selvmord skulle det være hevn for urett, men det finnes bedre alternativer. | **1** | |
| Hvis jeg gjorde selvmord, skulle det vekke berettiget oppmerksomhet hos andre. | **2** | |
| Hvis jeg gjør selvmord, løser det store problemer der jeg ikke har noe bedre alternativ. | **3** | |
| Et selvmord ville innebære en etterlengtet befrielse og hvile, både for meg og mine omgivelser. | **4** | |

| **20.** | |  |
| --- | --- | --- |
| Jeg verken tenker på eller planlegger selvmord. | **0** | |
| Jeg har iblant tenkt på selvmord. | **1** | |
| Jeg har iblant tenkt på ulike selvmordsmetoder, men jeg har ingen gjennomtenkte planer. | **2** | |
| Jeg har gjennomtenkte planer for å begå selvmord, men har ikke direkte gjort noen forberedelser. | **3** | |
| Jeg har alt klart for å gjøre selvmord og venter nå på en passende anledning. | **4** | |
